# Supplementary material for: Dopamine-Conjugated Carbon Dots Inhibit Human Calcitonin Fibrillation
Source: Nanomaterials (Basel). 2021 Aug 30;11(9):2242. doi: 10.3390/nano11092242 (PMC8465381; doi:10.3390/nano11092242)
Supplement: Supplementary file 1 [file nanomaterials-11-02242-s001.zip › nanomaterials-1351667-supplementary.pdf]

Supporting Information

Dopamine-Conjugated Carbon Dots Inhibit Human Calcitonin Fibrillation

Jhe-An Wu, Yu-Chieh Chen and Ling-Hsien Tu *

Department of Chemistry, National Taiwan Normal University, Taipei 106, Taiwan; xxxddd519@gmail.com (J.-A.W.); jj40344030s@gmail.com (Y.-C.C.)
* Correspondence: litu@gapps.ntnu.edu.tw

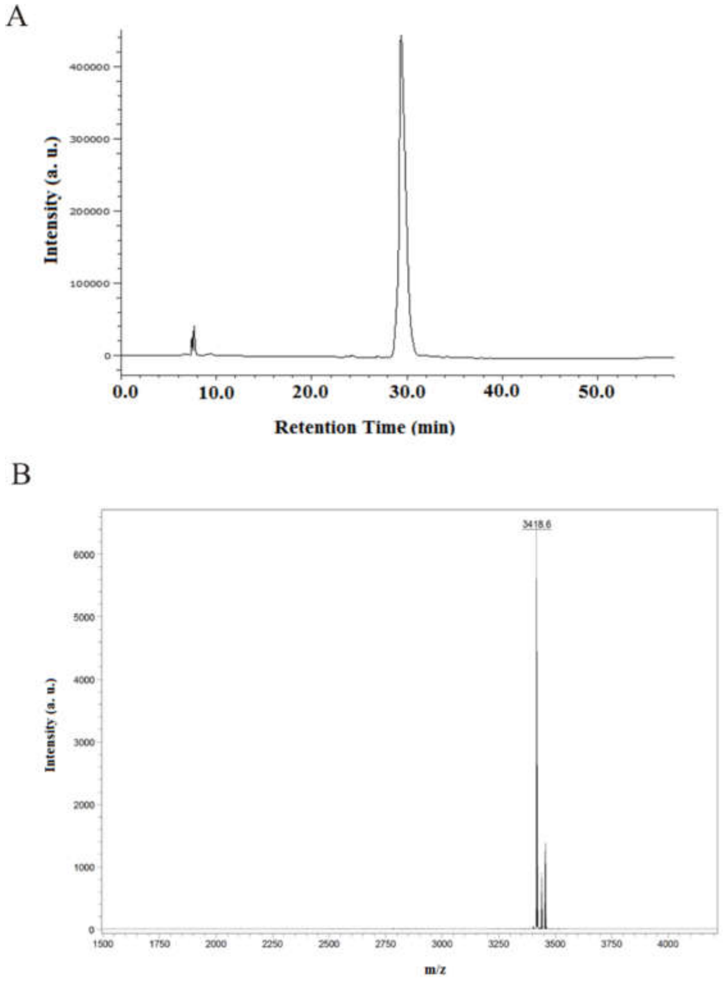

Figure S1. (A) HPLC chromatogram for hCT during 2nd purification. (B) MALDI spectrum for hCT ($C_{151}H_{226}N_{40}O_{45}S_3$) after purification. Calculated, 3417.85 m/z; measured, 3418.6 m/z.

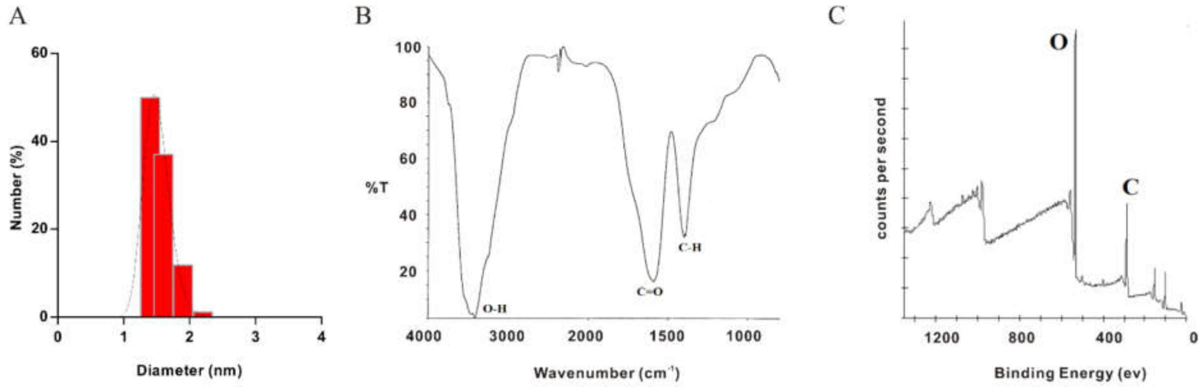

Figure S2. (A) Size distribution of CDs without any modification. (B) FTIR spectra of CDs. (C) XPS spectra of CDs.

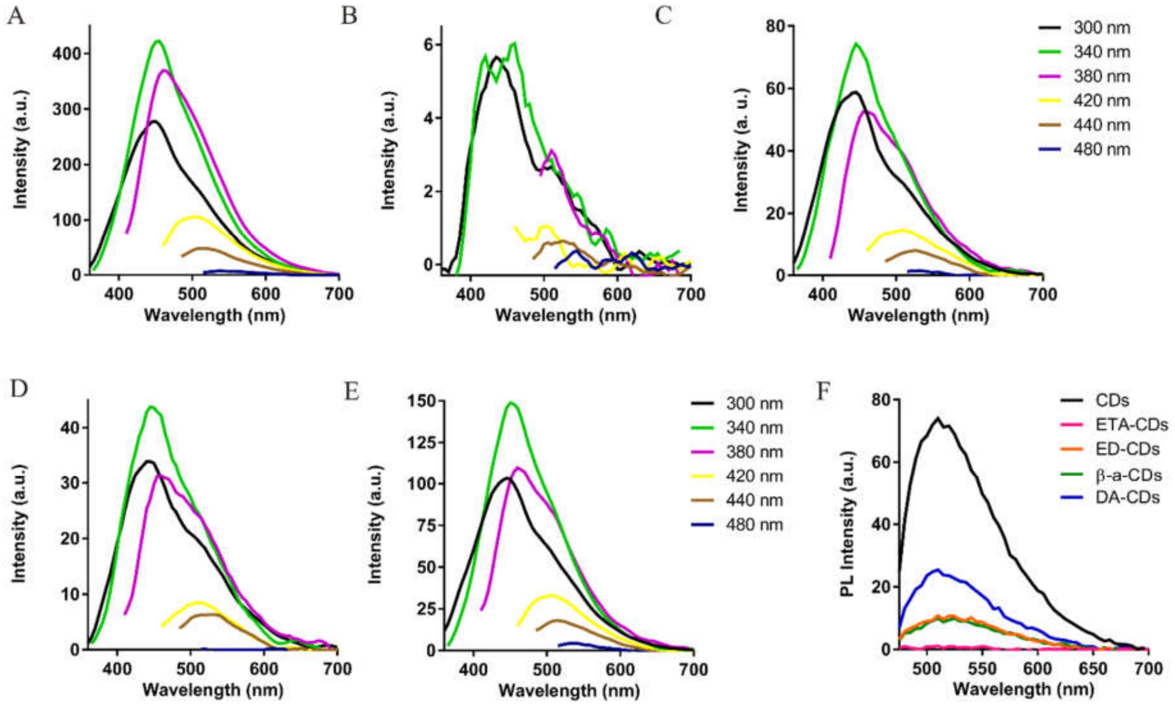

Figure S3. Emission spectra of (A) CDs, (B) ETA-CDs, (C) ED-CDs, (D) β-a-CDs, and (E) DA-CDs in DDI water excited at different wavelength. (F) Emission spectra of five carbon dots upon excited at 430 nm.

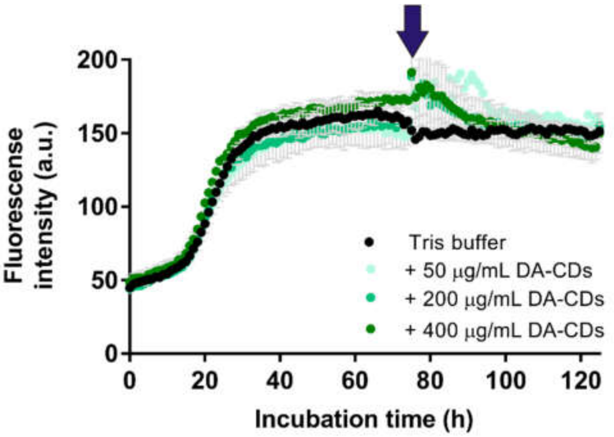

Figure S4. ThT monitored IAPP fibril dissociation by DA-CDs. DA-CDs were added at time point indicated by blue arrow.

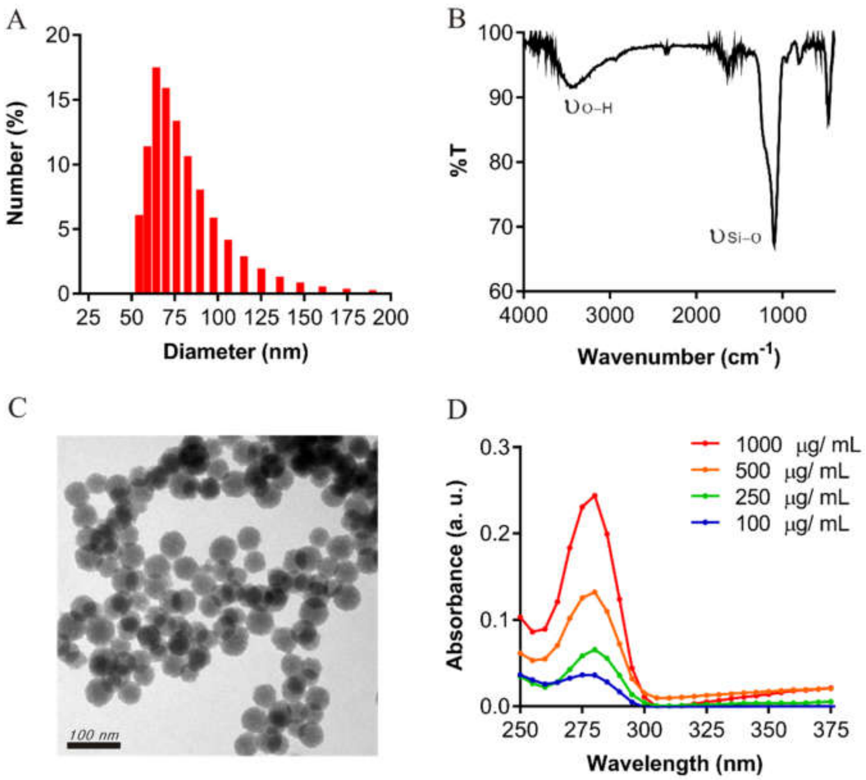

Figure S5. (A) Size distribution of DA-MSNs. (B) FTIR spectra of DA-MSNs. (C) TEM images of DA-MSNs. (D) Absorption spectra of DA-MSNs prepared in different concentration were recorded from 250~375 nm.
